# Global Burden of Esophageal Cancer and Its Risk Factors: A Systematic Analysis of the Global Burden of Disease Study 2019

**DOI:** 10.3390/life15010024

**Published:** 2024-12-28

**Authors:** Irena Ilic, Ivana Zivanovic Macuzic, Ana Ravic-Nikolic, Milena Ilic, Vesna Milicic

**Affiliations:** 1 Faculty of Medicine, University of Belgrade, 11000 Belgrade, Serbia; 2 Department of Anatomy, Faculty of Medical Sciences, University of Kragujevac, 34000 Kragujevac, Serbia; 3 Department of Dermatovenerology, Faculty of Medical Sciences, University of Kragujevac, 34000 Kragujevac, Serbia; 4 Department of Epidemiology, Faculty of Medical Sciences, University of Kragujevac, 34000 Kragujevac, Serbia

**Keywords:** esophageal cancer, burden, risk factors, trends

## Abstract

Background: Esophageal cancer is a major public health issue, yet risk factors for its occurrence are still insufficiently known. This study aimed to estimate the global burden of esophageal cancer and its risk factors. Methods: This ecological study presented the incidence, mortality, and Disability-Adjusted Life Years (DALYs) of esophageal cancer in the world. This study collected the Global Burden of Disease study data from 1990 to 2019. Trends in esophageal cancer burden were assessed using the joinpoint regression analysis and calculating the average annual percent change (AAPC). Results: Globally, in 2019, in both sexes and all ages, the ASR for the incidence of esophageal cancer was 6.5 per 100,000 and for mortality, 6.1 per 100,000. The global proportion of DALYs for esophageal cancer attributable to selected behavioral, metabolic, and dietary risk factors was similar in males and females: chewing tobacco (3.8% vs. 5.1%), diet low in fruits (10.1% vs. 12.6%), diet low in vegetables (3.3% vs. 4.6%), and high body mass index (18.8% vs. 19.3%). However, the proportion of DALYs for esophageal cancer attributable to smoking and alcohol use was 4–5 times higher in males than in females (50.1% vs. 11.3%, and 29.6% vs. 5.1%, respectively). From 1990 to 2019, a significant decrease in global trends in rates of DALYs for esophageal cancer attributable to smoking (AAPC = −1.6%), chewing tobacco (AAPC = −0.5%), alcohol use (AAPC = −1.0%), a diet low in fruits (AAPC = −3.1%), and a diet low in vegetables (AAPC = −3.6%) was observed, while a significant increase in trends was observed in DALYs rates for esophageal cancer attributable to a high body mass index (AAPC = +0.4%). Conclusions: More epidemiological research is needed to elucidate the relationship between esophageal cancer and certain risk factors and guide prevention efforts.

## 1. Introduction

According to the GLOBOCAN 2020 estimates, esophageal cancer ranked as the 7th most common cancer in males worldwide, with Asia recording the highest incidence rates [1]. Based on estimated mortality rates in 2020, esophageal cancer was the 5th leading cause of cancer-related deaths in males in the world and 9th in females [2]. In 2020, esophageal cancer was the number one most often diagnosed cancer in males in Bangladesh and the 2nd leading cancer diagnosed in females in Yemen and Bangladesh. Based on mortality rates in 2020, esophageal cancer was the leading cancer in both sexes together in Bangladesh. Globally, the area with the highest incidence and mortality of esophageal cancer extends from Northern Iran through Central Asia to Mongolia and China, which is termed the “Asian esophageal cancer belt” [3].

Esophageal cancer is among the deadliest malignancies, with a still low survival rate (with an overall five-year survival rate of about 20% for all stages combined) [1,4,5]. Despite improvements in surgical technology and systematic treatment, a poor outcome in patients with esophageal cancer worldwide is linked to unclear early symptoms and diagnosis of the disease at an advanced stage [6]. The two major histological types of esophageal cancer are squamous cell carcinoma (making up about 80% of all cases) and adenocarcinoma (making up less than 20% of all cases), with different etiological, epidemiological, and clinical peculiarities [7,8,9].

Over the last three decades, a decline in incidence and mortality from esophageal cancer has been recorded in the world [10,11]. But, at the same time, the total number of new cases and deaths from esophageal cancer has increased due to population growth and aging [1]. Although the data on the economic burden due to esophageal cancer across the countries during the past years were insufficient and poorly comparable, esophageal cancer still causes a heavy economic burden [12,13]. It is predicted that from 2013–2030 in China, direct medical expenditure is going to increase by 128.7% (from USD 33.4 to USD 76.4 billion), with costs being 2–3 times higher in men than in women [12].

Previous research showed that the geographic disparities in the burden of esophageal cancer could be due to differences in the prevalence of certain risk factors, namely smoking, alcohol consumption, obesity, and diet [14,15,16]. The decline in the incidence of esophageal cancer is related to the decrease in tobacco smoking, especially in developed Western countries [17]. Still, a clear understanding of the etiology of esophageal cancer is lacking. The Sustainable Development Goals, announced in 2015 by the United Nations and the World Health Organization, include target 3.4, which aims to reduce premature mortality attributable to cancer by one-third by 2030 [18].

In order to achieve this goal, it is important to better understand the patterns of esophageal cancer burden in the world because this can provide additional insights into the etiology and ways for more effective control of the disease. This study aimed to estimate the burden of esophageal cancer and its risk factors in order to improve the understanding of esophageal cancer as a meaningful public health issue.

## 2. Materials and Methods

### 2.1. Study Design

An epidemiological study (with an ecological study design) was carried out to describe the esophageal cancer burden worldwide over the 1990–2019 period and to evaluate the esophageal cancer burden attributable to selected risk factors.

### 2.2. Data Source

Data on esophageal cancer were collected from the Global Burden of Disease (GBD) 2019 study database (https://vizhub.healthdata.org/gbd-results/; accessed on 12 July 2023) [19]. The GBD study produces high-quality estimates of esophageal cancer burden around the world and also assesses the contribution of certain risk factors to the burden of esophageal cancer [19]. In order to provide these estimates, the GBD 2019 study obtained data from multiple sources, including national vital statistics, cancer registries, verbal autopsy reports, and national health surveys. Esophageal cancer includes malignant neoplasms defined by the International Statistical Classification of Diseases, Ninth Revision (ICD-9), as codes 150–150.9, or by the Tenth Revision (ICD-10) as codes C15–C15.9 [19,20].

The GBD 2019 study is compliant with the best reporting practices for studies that calculate health estimates, i.e., the Guidelines for Accurate and Transparent Health Estimates Reporting (GATHER) [21]. The GBD 2019 study reported data for esophageal cancer at the global and regional level (including 21 GBD regions and 5 Socio-demographic Index (SDI) quintiles), i.e., 204 countries and territories.

According to the GBD comparative risk assessment framework for determining the fraction of disease burden attributable to risk, some behavioral, dietary, and metabolic risk factors are associated with esophageal cancer (such as smoking, chewing tobacco, alcohol use, diet low in fruits, diet low in vegetables, and a high body mass index) [22]. The GBD risk factor hierarchy and accompanying exposure defined the exposure to a diet low in fruits, a diet low in vegetables, and a high body mass index (BMI) (>25 kg/m^2^) using the theoretical minimum risk exposure level as a reference value [19].

### 2.3. Study Variables and Measures

This study presents the esophageal cancer burden, including incidence, mortality, and Disability-Adjusted Life Years (DALYs). DALYs for esophageal cancer are calculated as the sum of the years of life lost due to premature mortality from that cause and the years of healthy life lost due to disability resulting from that cause. One DALY corresponds to the loss of the equivalent of one year of full health. For esophageal cancer burden, all figures were adjusted by age and presented as age-standardized rates (ASRs per 100,000 persons), calculated using the direct method of standardization according to the GBD 2019 standard population [19].

For countries/territories that had less than 100,000 inhabitants (such as Andorra, American Samoa, Antigua and Barbuda, Cook Islands, Dominica, Greenland, Marshall Islands, Monaco, Nauru, Niue, Northern Mariana Islands, Palau, Saint Kitts and Nevis, San Marino, Tokelau, and Tuvalu), due to the instability of the rates, the results are shown but were not considered in comparisons.

The Socio-demographic Index, as a summary measure of the level of development of a country, is a composite index based on three indicators: income per capita, average years of schooling in persons aged ≥ 15, and total fertility rate for females aged < 25 [19]. The SDI is expressed on a scale from 0.0 (lowest SDI) to 1.0 (highest SDI), with quintiles that categorize countries as low, low-middle, middle, high-middle, and high SDI.

### 2.4. Statistical Analysis

The direction and magnitude of esophageal cancer burden were evaluated using the joinpoint regression analysis software (Version 4.9.0.0; National Cancer Institute, Bethesda, Maryland, USA, March 2021), using the method proposed by Kim et al. [23]. In this analysis, for each model, a minimum of zero joinpoints (one line segment) and a maximum of five joinpoints (six line segments) were allowed. The Average Annual Percent Change (AAPC) over the entire observed period was calculated; for each AAPC estimate, its corresponding 95% confidence interval (95%CI) was computed [24]. With regards to the direction of trends, “significant change” (increase or decrease) was the term used to indicate that the slope of the trend was statistically significant (*p* < 0.05). Also, in this study, the global proportion (%) of ASRs of DALYs for esophageal cancer attributable to selected behavioral, dietary, and metabolic risk factors was presented.

Analysis was performed by sex, age, and region. The subgroup analyses by age were performed in five-year age groups from 0 to 95+ years. As there were very few cases of esophageal cancer reported among those under the age of 20 in most populations due to difficulties in computing due to unstable incidence/mortality/DALY rates, the results are not shown separately for ages < 20 years. Also, estimates were not produced for some risk factors in the age groups < 20, 20–24, and/or 25–29 years due to the GBD 2019 study modeling these risk factors with lower age restrictions of 30 years.

### 2.5. Ethical Considerations

This study was approved by the Ethics Committee of the Faculty of Medical Sciences, University of Kragujevac (Ref. No.: 01-14321, 13 November 2017), entitled “Epidemiology of the most common health disorders”. The data are fully aggregated, without any identification data, and no patient approvals were required for the study.

## 3. Results

**Figure 1:**
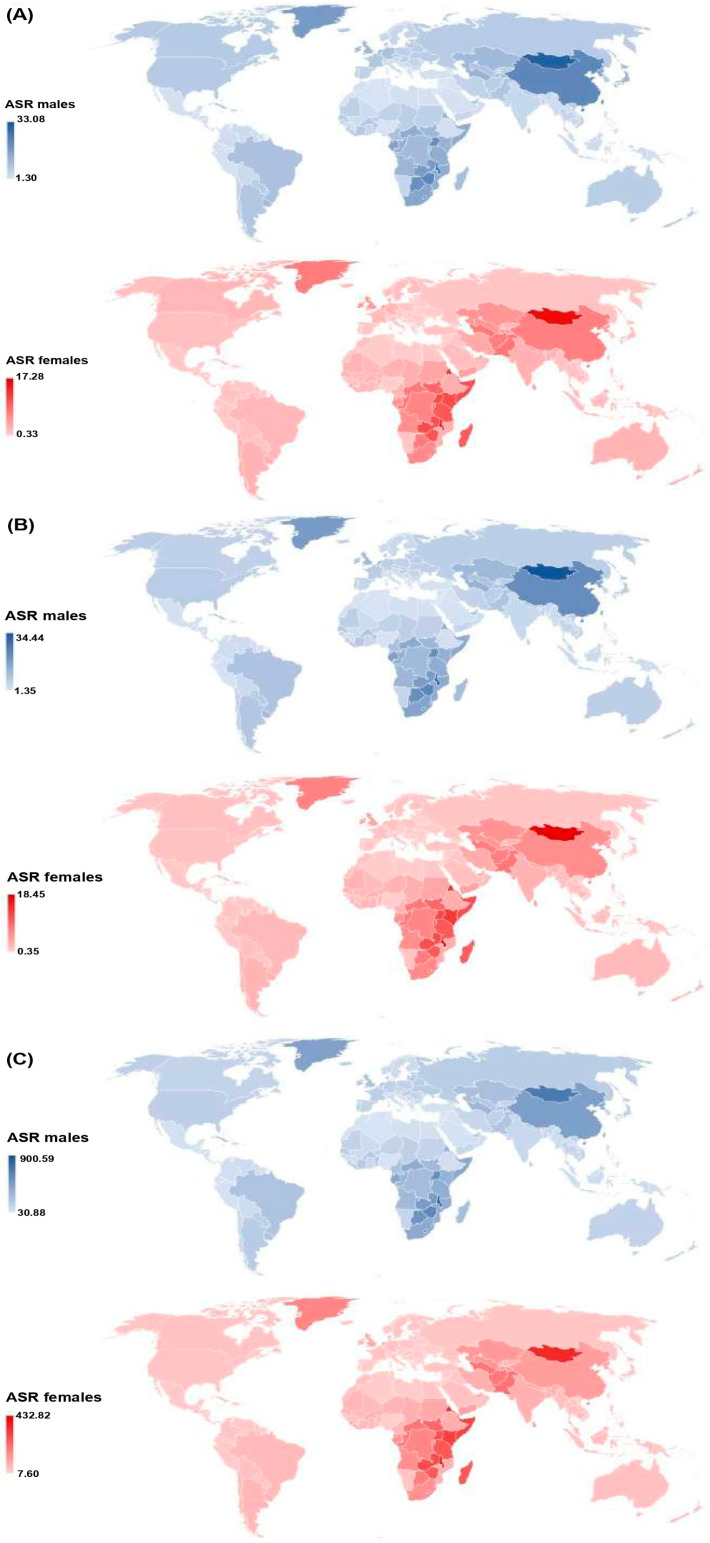
Age-standardized rates (ASRs) of incidence (**A**), mortality (**B**), and DALYs (**C**) of esophageal cancer across 204 countries/territories in 2019 by sex. ASR = Age-standardized rates (per 100,000); DALYs = Disability-Adjusted Life Years. Source: Global Burden of Disease study [19].

In 204 countries and territories, the total number of new cases of esophageal cancer was 534,563 (388,827 males and 145,736 females) in 2019 (Figure 1, Table 1). Most of the new cases (284,908; 53.2% of the total) were recorded in East Asia, and of those, the majority (278,121; 97.6%) were in China. In 2019, at the global level, the ASR of incidence of esophageal cancer was 6.5 per 100,000 in all ages and both sexes, while the male-female ratio was 3:1. The highest incidence ASRs (about 17.0 per 100,000) were found in Mongolia and Malawi, followed by some countries in Africa (Uganda, Kenya, Zambia, Somalia, Zimbabwe, Tanzania, Madagascar, and Rwanda) with ASRs of 10.0 per 100,000. The lowest ASRs (<0.5 per 100,000) were reported in Moldova, Belarus, Ukraine, Nigeria, Libya, and Tunisia.

Worldwide, the total number of esophageal cancer deaths in all ages and both sexes was 498,067 (365,554 males and 132,513 females) in 2019 (Figure 1, Table 1). Most of the deaths (257,316; 51.7% of the total) were recorded in China. At the global level, the ASR of mortality of esophageal cancer was 6.1 per 100,000 in both sexes, with a male-female ratio of 3:1. The highest mortality ASRs (nearly 19.0 per 100,000) were found in Mongolia and Malawi, followed by some countries in Africa (Eritrea, Kenya, and Uganda) with ASRs of about 13.0 per 100,000. The lowest ASRs (<0.5 per 100,000) were reported in Moldova, Ukraine, Nigeria, Libya, Tunisia, and the Republic of Korea.

In 2019, the total number of DALYs of esophageal cancer in all ages and both sexes was 11.7 million (Figure 1, Table 1). The highest number of DALYs was recorded in China (5.8 million; 49.6% of the total), followed by India (1.1 million; 9.4% of the total). The lowest number of esophageal cancer DALYs (<100) in 2019 was noted in North Macedonia and Micronesia. The highest ASR of DALYs of esophageal cancer was noted in Malawi (432.9 per 100,000), while the lowest rate was reported in the Republic of Korea and Moldova (equally 8.9 per 100,000).

**Figure 2:**
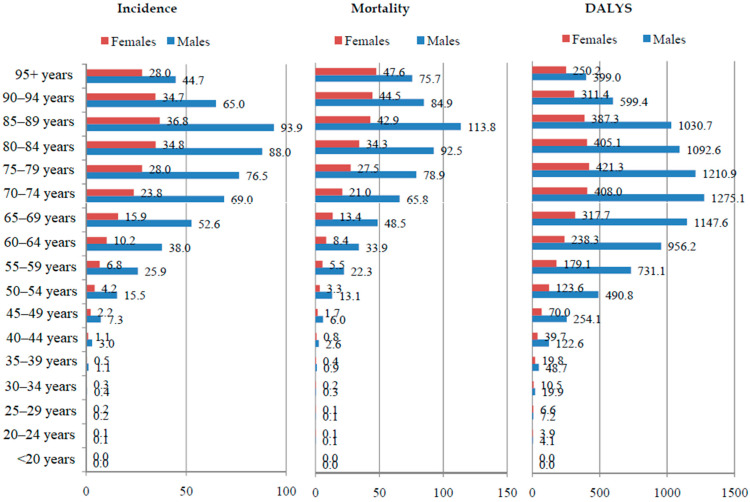
The global burden of esophageal cancer (incidence, mortality, and DALYs) rates (per 100,000 population) by age and sex in 2019. DALYs = Disability-Adjusted Life Years. Source: Global Burden of Disease study [19].

Global rates of incidence, mortality, and DALYs for esophageal cancer in males were always markedly higher than those in females in all age groups (especially in the 44–64 years age group) in 2019 (Figure 2).

The global ASRs of DALYs for esophageal cancer were decreasing worldwide both for males (AAPC = −1.1%, 95%CI = −1.4 to −0.8) and females (AAPC = −2.3%, 95%CI = −2.7 to −2.0) from 1990 to 2019; according to the comparability test, trends in males and females were parallel (*p* > 0.05) (Table 1). The ASRs of DALYs for esophageal cancer in both sexes together were decreasing in all SDI regions. However, only the GBD region of Western Sub-Saharan Africa showed a significant increase in the trend of ASRs for esophageal cancer for the observed period of 30 years (AAPC = +1.0%, 95%CI = 0.9 to 1.2), while the increase in ASRs for DALYs in the Caribbean region did not reach statistical significance (AAPC = 0.2%, 95%CI = −0.0 to 0.4).

**Figure 3:**
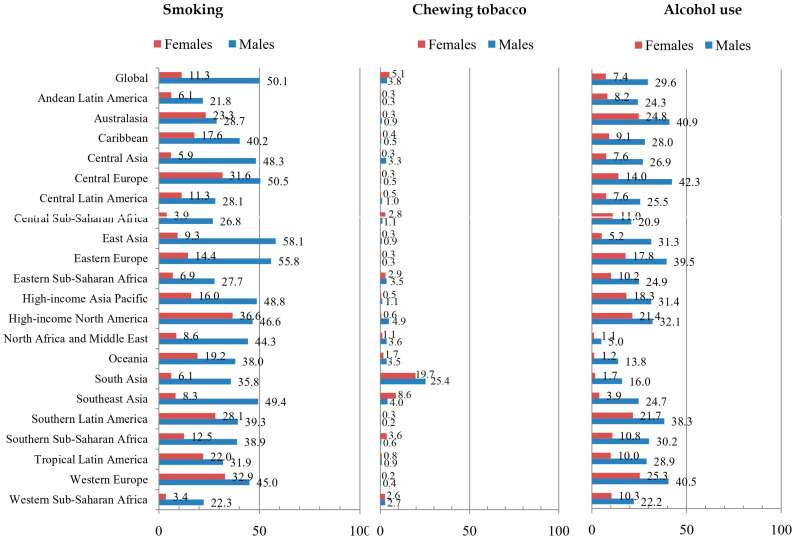
Proportion (%) of esophageal cancer (DALYs) attributable to behavioral risk factors for 21 GBD regions and globally, for all ages and both sexes together in 2019. DALYs = Disability-Adjusted Life Years; GBD = Global Burden of Disease. Source: Global Burden of Disease study [19].

Globally, the total proportion of DALYs for esophageal cancer attributable to smoking in all ages was 50.1% in males and 11.3% in females in 2019 (Figure 3). The highest proportion of DALYs was recorded among males in the regions of East Asia (58.1%) and Eastern Europe (55.8%) and among females in the high-income North America region (36.6%), while the lowest proportion was recorded among males in Andean Latin America (21.8%) and among females in Western Sub-Saharan Africa (3.4%). While the proportion of DALYs for esophageal cancer due to smoking was higher in males than in females in all GBD regions, the share of the global DALYs for esophageal cancer attributable to chewing tobacco was very heterogeneous by sex.

Globally, in 2019, the total proportion of DALYs for esophageal cancer attributable to chewing tobacco in all ages was 3.8% in males and 5.1% in females. The highest proportion of DALYs was recorded for both sexes in the South Asia region (25.4% in males, 19.7% in females), while the lowest proportion was recorded in males in Southern Latin America and in females in Western Europe (both equally 0.2%). The proportion of DALYs for esophageal cancer due to alcohol use was higher in males than in females in all GBD regions, whereby the global proportion of DALYs for esophageal cancer attributable to alcohol use in all ages was 29.6% in males and 5.1% in females in 2019. The highest proportion of DALYs was recorded for males in the region of Central Europe (42.5%) and for females in Western Europe (25.3%), while the lowest proportion was recorded both among males and females in North Africa and Middle East (1.1% and 5.0%, respectively).

**Figure 4:**
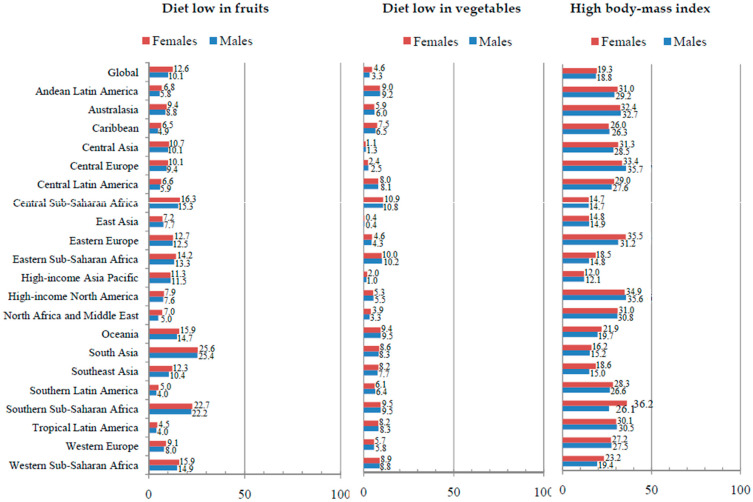
Proportion (%) of esophageal cancer (DALYs) attributable to behavioral and metabolic risk factors for 21 GBD regions and globally, for all ages and both sexes together in 2019. DALYs = Disability-Adjusted Life Years; GBD = Global Burden of Disease. Source: Global Burden of Disease study [19].

Globally, the total proportion of DALYs for esophageal cancer attributable to a diet low in fruits, a diet low in vegetables, and a high BMI in all ages was very similar in both sexes in 2019 (Figure 4). The total proportion of DALYs for esophageal cancer attributable to a diet low in fruits was 10.1% in males and 12.6% in females in 2019. The highest proportion of DALYs was recorded for both sexes in the region of South Asia (about 25.0%), while the lowest proportion was recorded in Tropical Latin America (about 4.0%). The total proportion of DALYs for esophageal cancer attributable to a diet low in vegetables was 3.3% in males and 4.6% in females in 2019. The highest proportion of DALYs was recorded for both sexes in the region of Sub-Saharan Africa (about 10.0%), while the lowest proportion was recorded in East Asia (0.4%). The total proportion of DALYs for esophageal cancer attributable to a high BMI was 18.8% in males and 19.3% in females in 2019. The highest proportion of DALYs was recorded for both sexes in the high-income North American region (about 35.0%), while the lowest proportion was recorded in the high-income Asia Pacific (about 12.0%).

**Figure 5:**
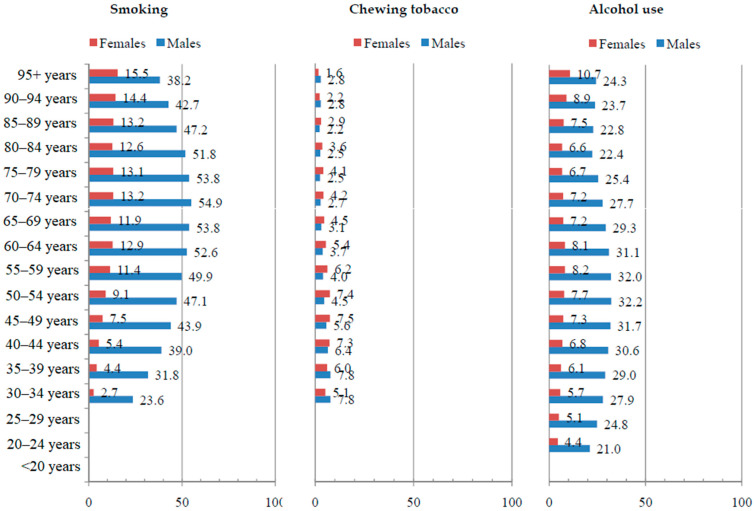
Proportion (%) of esophageal cancer (DALYs) attributable to behavioral risk factors globally by sex and by age in 2019. DALYs = Disability-Adjusted Life Years. Source: Global Burden of Disease study [19]. Note: Estimates were not produced for certain risk factors in the age groups <20, 20–24, and/or 25–29 years due to the GBD 2019 study modeling these risk factors with lower age restrictions of 30 years.

**Figure 6:**
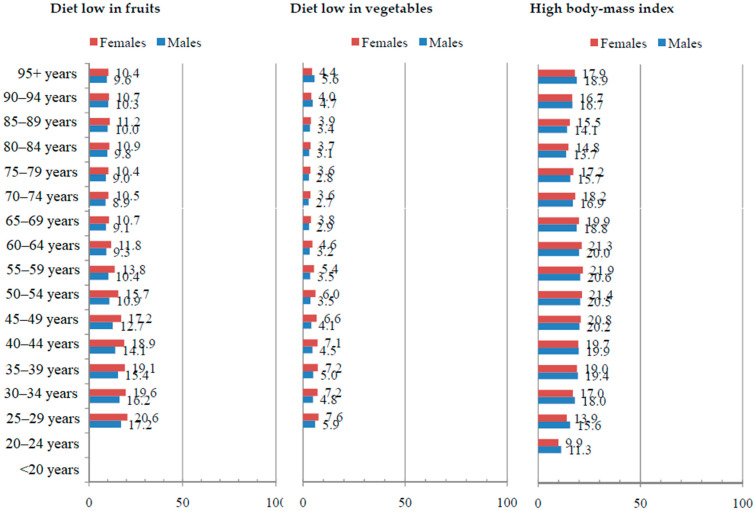
Proportion (%) of esophageal cancer (DALYs) attributable to behavioral and metabolic risk factors globally by sex and by age in 2019. DALYs = Disability-Adjusted Life Years. Source: Global Burden of Disease study [19]. Note: Estimates were not produced for certain risk factors in the age groups <20 and/or 20–24 years due to the GBD 2019 study modeling these risk factors with lower age restrictions of 30 years.

Globally, the total proportion of DALYs for esophageal cancer attributable to chewing tobacco, a diet low in fruits, and a diet low in vegetables was the highest in younger age groups and was very similar in males and females in 2019 (Figure 5 and Figure 6). The total proportion of DALYs for esophageal cancer attributable to smoking and alcohol use was 4–5 times higher in males than in females in all age groups, while the global proportion of DALYs for esophageal cancer attributable to a high BMI was very similar. While the proportion of DALYs for esophageal cancer attributable to smoking and alcohol use increased with age in females, in males, it was the highest in middle age.

**Figure 7:**
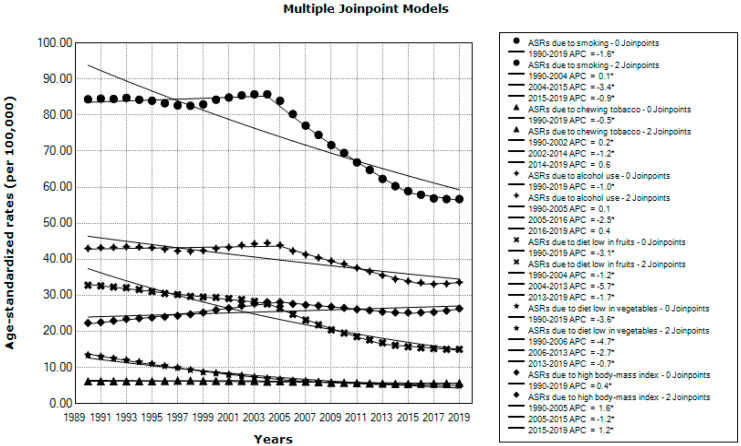
Trends in the global burden of esophageal cancer (DALYs) due to select risk factors, in all ages and both sexes together, in 1990–2019; a joinpoint regression analysis. ASR = Age-standardized rates (per 100,000); DALYs = Disability-Adjusted Life Years. * = Statistically significant trend (*p* < 0.05). Source: Global Burden of Disease study [19].

Significantly decreasing global trends in ASRs of DALYs for esophageal cancer attributable to smoking (AAPC = −1.6%), chewing tobacco (AAPC = −0.5%), alcohol use (AAPC = −1.0%), a diet low in fruits (AAPC = −3.1%), and a diet low in vegetables (AAPC = −3.6%) were observed from 1990 to 2019, while ASRs of DALYs for esophageal cancer attributable to a high BMI showed a significantly increasing trend (AAPC = +0.4%) (Figure 7).

## 4. Discussion

A decline in the global burden of esophageal cancer was observed in both sexes over the 1990–2019 period. Smoking and consumption of alcohol remained the major determinants of the global burden of esophageal cancer. Rates of DALYs for esophageal cancer attributable to smoking, chewing tobacco, alcohol use, a diet low in fruits, and a diet low in vegetables showed significantly decreasing global trends, while a significantly increasing trend was observed for global esophageal cancer burden attributable to a high body mass index. The magnitude of the burden of esophageal cancer attributable to risk factors varied between regions and by age and sex.

The burden of esophageal cancer shows remarkable variations across the world. In 2019, the patterns of esophageal cancer incidence, mortality, and DALYs across the world were very close, which reflects the very poor prognosis that characterizes this malignant tumor. In 204 countries and territories in 2019, the highest ASRs of incidence, mortality, and DALYs for esophageal cancer for both sexes together were recorded in Malawi and Mongolia, while the lowest rates were reported in Moldova; the differences were almost 50-fold. The GBD region of East Asia, followed by Southern Sub-Saharan Africa and Eastern Sub-Saharan Africa, exhibited the highest ASRs of incidence, mortality, and DALYs for esophageal cancer in 2019, while the lowest rates were found in the Andean Latin America region (10 times lower). There are two areas with the highest burden of esophageal cancer: the first, traditionally known as the Asian esophageal cancer belt, includes Eastern Asia and South-Central Asia, and the second, known as the African esophageal cancer corridor, includes Eastern Africa and Southern Africa [1,3]. In these two high-risk areas, about 80% of the world’s total cases of esophageal cancer occur, in which squamous cell carcinoma of the esophagus predominates [25,26]. On the other hand, esophageal adenocarcinoma is the dominant subtype in most of the highly developed countries in North America (the United States, Canada), Western and Northern Europe, and Australia [27,28,29]. Numerous epidemiological data linked significant geographical variations in incidence, mortality, and burden of esophageal cancer with medium/low socio-demographic index and metabolic risk factors such as high fasting plasma glucose, high low-density lipoprotein cholesterol, and a high body mass index [30,31]. A systematic review and meta-analysis of studies of esophageal cancer in Africa showed a strong association with tobacco and alcohol use and low socioeconomic status as risk factors [32]. Some reasons for the observed variations may be due to the differences in the age structure of populations in those areas, variations in socioeconomic development, availability and development of health services, variations in the diagnostic/treatment capacity between countries, as well as peculiarities of the cancer registries with differing data coverage and quality across countries [29,33].

The rates of esophageal cancer burden were about four times higher in men than in women, and the highest rates were observed among both sexes aged 65–80, declining thereafter. Rates of incidence, mortality, and DALYs for esophageal cancer in both sexes increased from the fifth decade of life, reaching a peak in the eighth decade, and then declined, with the exception of mortality in women, whose rates continuously increased with age. Bias due to decreasing diagnostic effort, lower rate of autopsies, or competing causes of death could hardly be linked to marked sex differences in esophageal cancer burden in the elderly. Previous research showed findings regarding the association between the female sex and a significant survival advantage of patients with esophageal cancer, whereby sex-based differences in survival significantly correlated with age, later menopause, and estrogen hormone replacement therapies [34]. Some authors have suggested that there might exist a protective effect of sex hormones in female patients with esophageal cancer, while others indicated that socioeconomic circumstances could have a role in the sex-dependent variations in the survival of esophageal cancer patients [35]. Contrary to that, a recent review showed an association between lower socioeconomic status, elderly females, lower income, squamous cell carcinoma of the esophagus, and refusal of esophagectomy with worse survival [36]. In addition, a possible explanation could be sought in the decreasing predominance of males due to the earlier appearance of esophageal adenocarcinoma as a consequence of exposure to obesity and/or gastroesophageal reflux, which are more common in men than in women at a younger age [37,38]. Although continued efforts should be made to elucidate this, the lag period must be taken into account, i.e., the influence of different phases of the epidemic process of esophageal cancers since the adoption of unhealthy habits (such as cigarette smoking, alcohol use, etc.) between males and females [16,17,39]. Additionally, the “low-high-low” trends of age-specific sex ratio in esophageal cancer burden indicate an effect of risk factors that are age-dependent and warrant further research to better understand the underlying etiologies of esophageal cancer.

There were apparent geographical differences regarding DALYs of esophageal cancer that could be due to selected behavioral, metabolic, and dietary risk factors in 2019. The proportions of DALYs for esophageal cancer attributable to cigarette smoking and chewing tobacco were the highest in Asia and attributable to an unhealthy diet in Africa and alcohol use in Europe; the proportion of DALYs for esophageal cancer due to a high body mass index was present in almost all of the developed areas in the world. Also, the present study found that the global proportion of DALYs for esophageal cancer was similar in males and females for chewing tobacco (3.8% in males and 5.1% in females), a diet low in fruits (10.1% vs. 12.6%), a diet low in vegetables (3.3% vs. 4.6%), and a high body mass index (18.8% vs. 19.3%), while the proportion of DALYs for esophageal cancer attributable to smoking and alcohol use was 4–5 times higher in men than in women (smoking: 50.1% in males and 11.3% in females; alcohol use: 29.6% in males and 5.1% in females). In this study, proportions of DALYs for esophageal cancer attributable to cigarette smoking and alcohol use were the highest in middle-aged males, while in females, they increased with age and were the highest in the oldest age. Also, the unfavorable burden of esophageal cancer in elderly females can be at least partially related to the fact that in younger males, the proportion for cigarette smoking was ten times higher and for alcohol use was five times higher compared to younger females, while those proportions are only twice as higher in men than in women in the oldest age. Apart from the abovementioned, the question remains regarding the proportion of the global esophageal cancer burden attributable to selected behavioral, metabolic, and dietary risk factors that could be explained by the variations among areas in enforced strategies of prevention, variations in genetic factors, and possible exposure to some not yet known factors [28,40,41]. It remains to be determined whether the effects of interaction in exposure to various factors (like smoking and alcohol use) on the esophageal cancer burden differ across populations around the world [42]. In Japan (a population with esophageal squamous cell carcinoma as the predominant form of esophageal cancer, with smoking and alcohol use as risk factors), genomic analysis determined germline polymorphisms in ALDH2 and CYP2A6, which affect alcohol and tobacco metabolism [43]. Also, a matched case-control study in a Chinese Han population reported that the polymorphism of CYP2C19*2 has an important role in the development of esophageal squamous cell carcinoma, modified by drinking tea and consuming pickled vegetables or hot beverages/food [44]. In Northeast Iran (an area with one of the highest esophageal squamous cell carcinoma rates in the world), the Golestan Cohort Study (throughout 10 years of follow-up of a population-based cohort of 50,045 individuals aged 40 to 75) revealed that nearly 75% of this region’s cases could be attributed to combined exposure to identified risk factors, including opium smoking, drinking hot tea, low intake of fruits and vegetables, excessive tooth loss, and exposure to indoor air pollution [45]. The International Agency for Research on Cancer classified alcohol use, betel, smoking, radiation, and a high body mass index as carcinogenic agents for esophageal cancer [46].

The global ASR of DALYs due to esophageal cancer declined in both sexes together from 1990 to 2019. Although it is not clear what the reasons are behind the decline in the burden of esophageal cancer across most of the GBD regions, there are some possible explanations that could help clarify this trend [47,48]. In this study, significantly decreasing global trends of ASRs of DALYs were observed for esophageal cancer attributable to almost all selected behavioral, metabolic, and dietary risk factors, while a significantly increasing trend was observed for esophageal cancer burden attributable to a high body mass index. A lot of research showed inconsistent results on the association between body mass index and esophageal cancer [25,49], with the pattern of association differing by subtype of esophageal cancer; a high body mass index is often associated with a lower risk for esophageal squamous cell carcinoma and with a higher risk for esophageal adenocarcinoma [50,51]. Although a negative correlation between a high body mass index and the risk of esophageal squamous cell carcinoma has been observed [25], a large retrospective nationwide cohort study in South Korea that comprised 22,809,722 persons reported that the relationship may be different; namely, abdominal obesity, as measured by waist circumference (defined as >90 cm for men and >85 cm for women), increased the risk for esophageal cancer [52]. In a recent updated meta-analysis, the assessed epidemiologic evidence regarding the causal relationship between body mass index and esophageal cancer indicated that obesity could increase the relative risk for esophageal adenocarcinoma (2.34; 95%CI = 2.02 to 2.70) while decreasing the risk for esophageal squamous cell carcinoma (0.71; 95%CI = 0.60 to 0.84) [15]. Even though the etiopathogenetic mechanisms behind the link between high body mass index and esophageal cancer are not completely clear, research suggests that altered gastroesophageal reflux disease and metabolic sequelae may be involved in esophageal carcinogenesis, involving molecular mechanisms such as increased expression of leptin receptors in esophageal adenocarcinoma and its direct proinflammatory effects on the epithelium of esophagus [53]. However, the molecular mechanisms are still not fully elucidated, and further research into the development of esophageal cancer is warranted. In the highly developed Western countries (like the United States, Canada, and Europe), in which the burden of esophageal adenocarcinoma was higher than the burden of esophageal squamous cell carcinoma, most of the esophageal cancer cases were caused by smoking, excessive drinking, obesity, and gastroesophageal reflux disease [54,55]. Over the last few decades, with improved socioeconomic status, better population health awareness, and the tobacco control program, the decline in the prevalence of smoking, in particular, affected the decline in esophageal cancer burden [55,56]. In Thailand, some studies revealed an association between poor oral health, Campylobacter infection, and infection with the Human papillomavirus and esophageal cancer risk [57,58]. In Malawi, as well as in other countries in Sub-Saharan Africa with the highest burden of esophageal cancer, although risk factors for this cancer are poorly understood, some exposures were reported, including low socioeconomic status, alcohol use, poor oral health (oral bacteria Fusobacterium and Prevotella), smoking, HIV+ status, drinking, mold on stored grain, and an estimated lower supply of Fe, Mg, Zn, and Se in the diet [32,59]. In addition, the overall survival for esophageal squamous cell carcinoma, which represents >90% of all esophageal cancer in Malawi, was very poor, likely due to the advanced disease stage and limited treatment access [60]. Consequently, continued etiologic studies to identify potential opportunities for prevention at a population level are critical to decreasing the burden of esophageal cancer around the world. There is a need for more research on the epidemiology and clinical features of esophageal cancer, particularly from the contemporary standpoint of population growth and aging, and with the disease still demonstrating a very poor prognosis. Also, despite the existing efforts for screening, more optimized and more successful modalities are needed to enable early diagnosis and improve survival [49]. Findings on the disparities in the burden of esophageal cancer worldwide and the contribution of different risk factors are necessary for providing directions on the development and implementation of tailored and region-specific screening practices and strategies for early detection and diagnosis of this disease, including the application of artificial intelligence in analysis of findings of imaging techniques which has the potential to improve disease outcomes [61]. Also, a comprehensive patient-centered approach to care is important, with supportive care intervention aimed at improving the quality of life, which, according to research, could reduce the risk of mortality from esophageal cancer [62].

### 4.1. Implications for Public Health Policy

In summary, the apparent geographic differences in the burden of esophageal cancer and in the contribution of certain risk factors indicate the need for the implementation of organized public health measures for prevention and disease control worldwide. Considering the fact that identified risk factors are modifiable, primary prevention represents a key factor in reducing the esophageal cancer burden. To achieve this, it is necessary to adopt a more effective approach to prevention and early diagnosis of esophageal cancer, along with continued implementation of a healthy lifestyle (especially tobacco control, reduction of alcohol use, and obesity prevention). The body mass index should be consistently checked and controlled, with the promotion of activities related to maintaining a healthy body weight and increasing physical activity. It should be emphasized that persons who already have an increased body mass index and have a higher risk for gastro-esophageal reflux or Barrett’s esophagus should particularly be targeted with interventions aimed at reducing weight such as offering spaces and opportunities for recreation activities. Interventions aimed at increasing the intake of fruits and vegetables should particularly target settings with lower resources and persons with lower income and educational levels. Hence, public health officers should develop strategies to promote a diet that is well-balanced in countries of lower SDI quintiles. Populations and regions of the world where alcohol consumption is more prevalent should be targeted with prevention efforts, especially among heavy drinkers. This, as well as smoking, should be the focus of interventions that would involve setting up psychological and counseling support systems, in addition to establishing laws and taxes related to obtaining and consuming these harmful substances. Public health stakeholders should develop strategies for increasing public awareness of the importance of a healthy lifestyle through education about risk factors, with the goal of mitigating the risk for esophageal cancer, reducing its burden, and thus contributing to the Sustainable Development Goal 3 target of reducing the deaths from non-communicable diseases by one third by 2030 [63].

### 4.2. Strengths and Limitations

This study evaluated global patterns of esophageal cancer burden from 1990 to 2019, as well as the participation of certain risk factors in the burden of esophageal cancer in the world. Further, this study reported estimates of esophageal cancer burden for 204 countries and territories, applying high-quality data from the GBD 2019 study. Additionally, the joinpoint analysis allows us to interpret the magnitude and direction of temporal trends and to establish whether those changes had statistical significance. Still, there are sources of limitations of this study that should be considered. Firstly, the question of the quality of esophageal cancer data always exists. Furthermore, the limitation of the study is the impossibility of a more detailed analysis because the data regarding the histological subtype of esophageal cancer is lacking. Additionally, future burden estimates should be strengthened by factoring in the combined effects of risk exposures in an attempt to capture potential synergy between some risk factors. Further on, due to the issue of under-reporting of data on esophageal cancer and exposure to risk factors, particularly in developing countries, the presence of bias cannot be entirely eliminated. Thus, this study investigated the selected risk factors that were considered and available in the GBD 2019 study, so it should be noted that careful consideration of all possible risk factors for esophageal cancer occurrence (i.e., genetic predisposition, environmental exposure, socioeconomic determinants, etc.) should be important for future research as knowledge expands and data availability increases. Finally, an ecological fallacy (i.e., fallacy inherent in making inferences regarding individual-level associations based on population-level data) that is inherent to the study design represents another relevant limitation of this study. Consequently, the findings that resulted from the correlation without adjusting for confounders should be very carefully interpreted. Thus, the findings of this study regarding the link between the burden of esophageal cancer and risk factors must be further elucidated in analytical longitudinal studies.

## 5. Conclusions

Esophageal cancer burden is among the major public health concerns in the world. There are apparent international variations in the esophageal cancer burden attributable to selected behavioral, metabolic, and dietary risk factors. Strategies for organized implementation of more effective preventive measures for esophageal cancer, which will aim to find further opportunities to address exposure to identified modifiable risk factors, are needed and require attention and effort from health professionals worldwide. Further analytical epidemiological studies are also required to elucidate the relationship between esophageal cancer and certain risk factors.

## Figures and Tables

**Table 1:** Burden of esophageal cancer, in all ages, by locations, 1990–2019; a joinpoint analysis.

Locations	Incidence in 2019		Deaths in 2019		DALYs in 2019		AAPC, of DALYs (ASRs), (95%CI)
	Cases	ASR	Cases	ASR	Cases	ASR	
Global							
Male	388,827	10.1	365,554	9.7	8,821,716	221.4	−1.1 * (−1.4 to −0.8)
Female	145,736	3.3	132,513	3.0	2,844,300	65.3	−2.3 * (−2.7 to −2.0)
Both sex	534,563	6.5	498,067	6.1	11,666,017	139.8	−1.4 * (−1.7 to −1.1)
— SDI regions							
High SDI	95,911	5.2	79,088	4.2	1,653,972	95.8	−0.6 * (−0.7 to −0.6)
High–middle SDI	145,268	7.1	135,757	6.6	3,105,596	151.0	−1.2 * (−1.5 to −0.9)
Middle SDI	205,238	8.4	193,720	8.2	4,485,644	175.2	−2.4 * (−2.9 to −1.9)
Low–middle SDI	59,864	4.4	60,670	4.5	1,611,655	111.3	−0.7 * (−0.8 to −0.6)
Low SDI	28,132	5.4	28,684	5.7	805,543	141.1	−0.5 * (−0.6 to −0.5)
— GBD regions							
Andean Latin America	827	1.5	889	1.6	18,839	33.4	−1.1 * (−1.2 to −1.0)
Australasia	2192	4.4	2035	4.0	39,885	85.2	−0.5 * (−0.6 to −0.4)
Caribbean	1920	3.7	1923	3.7	47,316	90.7	0.2 (−0.0 to 0.4)
Central Asia	4834	6.7	4924	7.1	129,818	164.8	−3.0 * (−3.2 to −2.8)
Central Europe	5853	2.9	5856	2.9	143,701	74.7	−0.4 * (−0.5 to −0.2)
Central Latin America	3869	1.7	4021	1.7	90,775	38.0	−1.5 * (−1.7 to −1.4)
Central Sub-Saharan Africa	4431	8.4	4509	9.0	127,510	215.2	−1.2 * (−1.3 to −1.1)
East Asia	284,908	13.7	263,307	13.0	5,922,865	275.4	−2.2 * (−2.7 to −1.7)
Eastern Europe	11,086	3.2	10,655	3.1	277,541	83.5	−1.5 * (−1.8 to −1.2)
Eastern Sub-Saharan Africa	16,391	10.0	16,940	10.8	476,744	263.4	−0.5 * (−0.6 to −0.4)
High-income Asia Pacific	25,159	5.7	16,337	3.5	306,118	75.8	−1.8 * (−1.9 to −1.6)
High-income North America	26,162	4.2	24,152	3.8	524,630	88.3	−0.1 * (−0.2 to −0.0)
North Africa and Middle East	10,024	2.4	9968	2.4	259,488	55.6	−0.7 * (−0.7 to −0.6)
Oceania	147	2.2	147	2.3	4213	53.6	−0.1 * (−0.1 to −0.1)
South Asia	53,488	3.8	54,161	3.9	1,476,590	98.3	−0.8 * (−1.0 to −0.7)
Southeast Asia	15,543	2.5	15,330	2.6	403,725	61.7	−0.6 * (−0.6 to −0.5)
Southern Latin America	3945	4.7	4067	4.8	83,206	101.1	−2.2 * (−2.4 to −2.1)
Southern Sub-Saharan Africa	5941	10.7	6095	11.3	159,882	267.1	−1.6 * (−2.2 to −1.0)
Tropical Latin America	12,684	5.2	12,767	5.2	328,430	131.0	−0.9 * (−1.0 to −0.9)
Western Europe	40,174	4.6	34,847	3.9	706,817	88.6	−1.0 * (−1.1 to −0.9)
Western Sub-Saharan Africa	4986	2.7	5135	2.9	137,923	67.8	+1.0 * (0.9 to 1.2)

ASR = Age-standardized rates (per 100,000); For full period (1990–2019) presented AAPC = Average Annual Percentage; Change; 95% CI = Confidence Interval; SDI = Socio-demographic Index. * Statistically significant trend (*p* < 0.05). Source: Global Burden of Disease study [19].

## Data Availability

Data are contained within the article.
